# Deregulation of miRNAs in malignant pleural mesothelioma is associated with prognosis and suggests an alteration of cell metabolism

**DOI:** 10.1038/s41598-017-02694-0

**Published:** 2017-06-09

**Authors:** Chiara De Santi, Ombretta Melaiu, Alessandra Bonotti, Luciano Cascione, Gianpiero Di Leva, Rudy Foddis, Alfonso Cristaudo, Marco Lucchi, Marco Mora, Anna Truini, Andrea Tironi, Bruno Murer, Renzo Boldorini, Monica Cipollini, Federica Gemignani, Pierluigi Gasparini, Luciano Mutti, Stefano Landi

**Affiliations:** 1 Respiratory Research Division, Department of Medicine, Education and Research Centre, Royal College of Surgeons in Ireland, Beaumont Hospital, Dublin 9, Ireland; 2 Immuno-Oncology Laboratory, Department of Paediatric Haematology/Oncology, Ospedale Pediatrico Bambino Gesù, Viale di S. Paolo 15, 00146 Rome, Italy; 3 Preventive and Occupational Medicine, University Hospital of Pisa, Pisa, Italy; 4 Lymphoma and Genomics Research Program, Institute of Oncology Research, Bellinzona, Switzerland; 5 School of Environment and Life Sciences, University of Salford, Manchester, United Kingdom; 6 Department of Translational Research and of new Technologies in Medicine and Surgery, University of Pisa, Pisa, Italy; 7 Division of Thoracic Surgery, Cardiac and Thoracic Department, University of Pisa, Pisa, Italy; 8 IRCCS H, San Martino-IST Genova, Genova, Italy; 9 Section of Anatomic Pathology, Oncology and Experimental Immunology, Department of Molecular and Translational Medicine, University of Brescia, Brescia, Italy; 10 Azienda ULSS 12 Veneziana, Venice, Italy; 11 Department of Health Sciences, School of Medicine, University Hospital Maggiore della Carità, Novara, Italy; 12 Department of Biology, University of Pisa, Pisa, Italy; 13 Department of Molecular Virology, Immunology and Medical Genetics, Ohio State University Wexner Medical Center and Comprehensive Cancer Center, Columbus, Ohio USA

## Abstract

Malignant pleural mesothelioma (MPM) is an aggressive human cancer and miRNAs can play a key role for this disease. In order to broaden the knowledge in this field, the miRNA expression was investigated in a large series of MPM to discover new pathways helpful in diagnosis, prognosis and therapy. We employed nanoString nCounter system for miRNA profiling on 105 MPM samples and 10 healthy pleura. The analysis was followed by the validation of the most significantly deregulated miRNAs by RT-qPCR in an independent sample set. We identified 63 miRNAs deregulated in a statistically significant way. MiR-185, miR-197, and miR-299 were confirmed differentially expressed, after validation study. In addition, the results of the microarray analysis corroborated previous findings concerning miR-15b-5p, miR-126-3p, and miR-145-5p. Kaplan-Meier curves were used to explore the association between miRNA expression and overall survival (OS) and identified a 2-miRNA prognostic signature (Let-7c-5p and miR-151a-5p) related to hypoxia and energy metabolism respectively. *In silico* analyses with DIANA-microT-CDS highlighted 5 putative targets in common between two miRNAs. With the present work we showed that the pattern of miRNAs expression is highly deregulated in MPM and that a 2-miRNA signature can be a new useful tool for prognosis in MPM.

## Introduction

MicroRNAs (miRNAs) are highly conserved small non-coding RNA molecules, 20–25 nucleotides long, which play an important regulatory role at post-transcriptional level. Each miRNA has multiple targets, thus slight variations in their expression could affect the behavior of a large variety of genes. Their abnormal expression has been linked to multiple human diseases, including cancer^1^. MiRNAs can function as either tumour suppressors or oncogenes and this feature, together with their tissue-specific expression, has prompted the study of miRNAs expression levels as a possible important diagnostic and prognostic tool for several malignancies^2^. Moreover, miRNAs-driven genes/pathways deregulation gives countless potentially druggable targets for the development of innovative cures^3^.

In recent years a number of studies have profiled the miRNA content of Malignant Pleural Mesothelioma (MPM) cell lines and/or tumour tissues^4^. MPM is a lethal cancer with increasing worldwide incidence, often induced by asbestos exposure. To date, a few studies have evaluated the differential expression of miRNAs using different sample sources in the hope of improving MPM management. Recently, Truini *et al*. reviewed a wide range of publications regarding MPM miRNA expression data^4^ highlighting the heterogeneity of the results (sometimes contradictory) especially due to ‘technical factors’ such as: diversity of the histological subtypes examined, different sample sources, diversity of the control groups, statistical approaches and different high-throughput platform used. From the published literature it seems established that miRNAs deregulated in MPM target genes fundamental for the development and progression of the disease, such as *CDKN2A*, *NF2*, *JUN*, *HGF*, and *PDGFA* ^4^. As a matter of fact, most of these miRNAs mapped to chromosomal regions known to be altered in MPM, such as gain of 8q24 and deletion of 1p36, and 14q32.

To overcome some conflicting results, larger samples’ cohorts should be profiled and analysed to minimize false-positive findings. In order to broaden the knowledge on the miRNAs expression status in MPM, we profiled a large number of histologically confirmed FFPE MPM samples, using NanoString nCounter, a high throughput platform particularly suitable for miRNA profiling of FFPE samples. Validation of the most significantly deregulated miRNAs was done by the gold standard quantitative RT-qPCR on an independent group of MPM and controls tissues. Furthermore, using *in silico* approaches, we sought to better understand how the biology of MPM might be differentially impacted by the expression patterns of key miRNAs. Finally, miRNA expression was correlated to patient overall survival (OS). As a result, we built a 2-miRNA signature able to divide MPM patients in high and low risk that has allowed us to highlight the role of microenvironment and energy metabolism in MPM.

## Results

### Several miRNAs are deregulated in MPM

We used a microarray approach to analyse miRNAs profile of a series of 96 MPM patients compared to 10 normal pleura samples. Raw and normalised expression data are provided individually for each sample for each of the 800 miRNAs detected by the nanoString platform in Supplementary Table 1.

**Figure 1:**
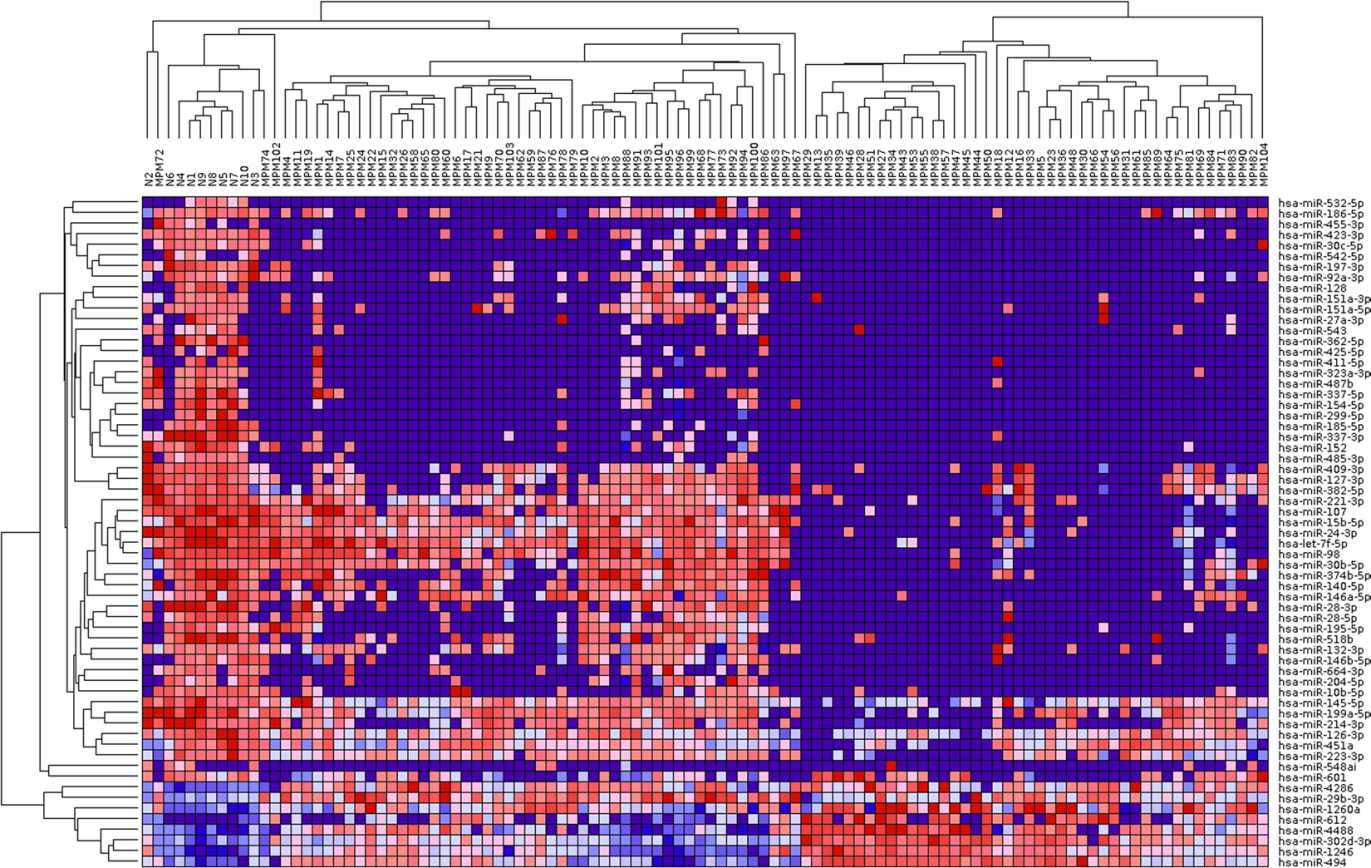
Heat map representing hierarchical clustering of 63 statistically significantly deregulated miRNAs detected in the present work on 96 MPM and 10 control tissues. Rows: miRNAs; columns: samples; red: high expression; blue: low expression.

Limma analysis identified a total of 63 deregulated miRNAs represented in Fig. 1. Among them, 55 were down-regulated and 8 were up-regulated in MPM samples (detailed information are available in Supplementary Table 2). The expression levels of miRNAs were ranked according to BH values and the top five significant miRNAs were: miR-337-3p, miR-185-5p, miR-485-3p, miR-197-3p, and miR-299-5p, all down-regulated in MPM. These were selected for validation with RT-qPCR in an independent cohort of 16 MPM and 17 control tissues. The extent of differential expression was quantified by calculating the log_2_(fold change) (i.e., log_2_(FC)) using the normal pleural specimens as control samples. We observed a statistically significant down-regulation in MPM samples, after Bonferroni’s correction, for miR-197, miR-185 and miR-299 (see Table 1), whereas we could not validate the results for miR-337 and miR-485. Raw and normalised Ct (Threshold Cycle) values for each sample for each miRNA are provided in Supplementary Table 3.

**Table 1:** Statistical analyses testing differences in expression levels (expressed as logarithm base 2 of the fold change) between MPM and controls in the validation series. In bold the three differentially expressed miRNAs are highlighted.

microRNA	Log_2_(FC)	p-value
**miR-197-3p**	−2.540	**3.72 × 10** ^**−7**^
**miR-185-5p**	−2.742	**2.22 × 10** ^**−4**^
**miR-299-5p**	−1.239	**0.0014**
miR-337-3p	−0.758	0.0201
miR-485-3p	−0.488	0.1886

In addition, from our microarray analysis we could confirm previous findings from several high-throughput studies where mesothelial cell lines or normal mesothelial tissues were used as controls (Table 2). Interestingly, we found a statistically significant down-regulation of miR-126-3p, miR-15b-5p, and miR-145-5p, which showed to have a functional role in MPM according to *in vitro* and *in vivo* studies^5–7^. The log_2_ (FC) was −0.307 for miR-126 (p-value after BH correction = 0.014), −0.917 for miR-15b (0.00265), and −0.326 for miR-145 (0.014). Thus, overall, we confirmed the role played by miR-126, miR-15b and miR-145 and, in addition, we suggest a role for miR-185, miR-197, and miR-299 in MPM.

**Table 2:** List of miRNAs deregulated according to the present nanostring analysis that were previously reported in relation to miRNA profiling data in MPM; BH = P-value of the comparison between MPM vs non-MPM after Benjamini-Hochberg correction for multiple test; the trend of the deregulation according to our analysis is expressed as “Differential Expression”, the trend reported in literature is expressed as arrows (↑ = up-regulated, ↓ = down-regulated).

miRNA	BH	Differential Expression	Direction of deregulation	References
hsa-miR-152	2,66 × 10^−12^	−2,31	↓	20
hsa-miR-30c-5p	1,73 × 10^−09^	−2,40	↓, ↑	20, 22
hsa-miR-542-5p	3,66 × 10^−08^	−0,30	↑	20
hsa-miR-92a-3p	5,26 × 10^−07^	−2,61	↑	21
hsa-miR-423-3p	2,28 × 10^−06^	−1,85	↓	19
hsa-miR-214-3p	5,75 × 10^−06^	−4,23	↓	21
hsa-miR-29b-3p	1,49 × 10^−05^	3,15	↓	20
hsa-miR-10b-5p	0,000144	−2,84	↓	20
hsa-miR-146b-5p	0,000298	−2,77	↓	21
hsa-miR-127-3p	0,000302	−4,89	↓	20
hsa-miR-221-3p	0,000633	−2,69	↓	20
hsa-miR-15b-5p	0,00265	−2,79	↓	6
hsa-miR-195-5p	0,00322	−2,53	↓	6
hsa-miR-204-5p	0,00399	−1,65	↓	7
hsa-miR-145-5p	0,01436	−1,83	↓	7, 23
hsa-miR-126-3p	0,01436	−1,76	↓	22, 23
hsa-miR-451a	0,04051	−2,63	↓	23
hsa-miR-146a-5p	0,04636	−2,93	↓	20

### *In silico* analysis suggested targets and pathways affected by the deregulated miRNAs

The putative mRNAs targeted by the 6 mentioned miRNAs (i.e. miR-126, miR-15b, miR-145, miR-185, miR-197 and miR-299) were predicted with DIANA-microT-CDS *in silico* tool and the top 50 predicted targets according to miTG score are reported in Supplementary Table 4. Interestingly, the intersection of the 6 lists of putative targets (shown in the last sheet of Supplementary Table 4) highlighted 5 transcripts in common between two miRNAs (*GOLGA1, GOLGB1, PTPN9, PSMD1, TNRC6A*).

When miRNAs were analysed with miRPath tool, the union of predicted or experimentally validated targets showed an enrichment for pathways involved in cancer and signaling cascades such as Wnt or MAPK pathways. The top 5 pathways significantly enriched are reported in Table 3, together with the miRNAs and their target genes mapped in the pathway. On the other hand, the intersection of predicted or experimentally validated targets highlighted the presence of further transcripts targeted by two or more deregulated miRNAs: *MPLZ1* (targeted by miR-145, -185, and -197), *PRKAR2A* (miR-185, -15b and -299), *CCNE1* (miR-15b and miR-185), and *VEGFA* (miR-126 and miR-15b).

**Table 3:** The topfive deregulated pathways detected by miRPath (using either TarBase or microT-CDS mode), based on the target genes of the 6 miRNAs resulted deregulated in a statistically significant way.

TarBasemode			
KEGG pathways	miRNAs mapped in the pathway	MiRNA target genes mapped in the pathway	Adjusted P-value
Pathways in cancer	miR-15b-5p, miR-126-3p, miR-145-5p, miR-299-5p, miR-197-3p, miR-185-5p	*E2F1, CRK, RAD51, PIK3R2, BCL2, IGF1R, RHOA, CDK6, TPM3, CCND1, CNE2, MMP1, AKT1, MYC, CDC42, CCNE1, CDKN1A, STAT1, VEGFA*	5.71 × 10^−13^
Hepatitis B	miR-15b-5p, miR-126-3p, miR-145-5p, miR-299-5p, miR-197-3p, miR-185-5p	*E2F1, ATF6B, PIK3R2, IFNB1, BCL2, CDK6, CCND1, CCNE2, AKT1, TIRAP, MYC, CCNE1, CDKN1A, STAT1*	2.10 × 10^−11^
Pancreatic cancer	miR-15b-5p, miR-126-3p, miR-145-5p, miR-197-3p, miR-185-5p	*E2F1, RAD51, PIK3R2, CDK6, CCND1, AKT1, CDC42, STAT1, VEGFA*	2.06 × 10^−09^
Small cell lung cancer	miR-15b-5p, miR-126-3p, miR-145-5p, miR-185-5p	*E2F1, PIK3R2, BCL2, CDK6, CCND1, CCNE2, AKT1, MYC, CCNE1*	9.77 × 10^−09^
Non-small cell lung cancer	miR-15b-5p, miR-126-3p, miR-197-3p, miR-185-5p	*E2F1, PIK3R2, CDK6, CCND1, AKT1, FOXO3*	7.40 × 10^−08^
**microT-CDS mode**			
Fatty acid biosynthesis	miR-15b-5p, miR-185-5p	*FASN, OXSM, ACACB*	5.48 × 10^−23^
MAPK signaling pathway	miR-15b-5p, miR-145-5p, miR-299-5p, miR-197-3p, miR-185-5p	*CACNG8, PDGFRA, CACNA2D3, NFKB1, PTPRQ, GNA12, SOS2, CRK, MAPK7, PAK2, GCK, CACNB4, CACNG7, MAPK14, LAMTOR3, RAF1, MAP3K4, TAOK1, MAP2K6, IKBKB, FGF11, PPP3CA, NLK, DUSP10, RAPGEF2, NFATC2, MAPK8, PPM1A, FLNB, FLNA, CACNA1E, CACNG4, FGF2, CDC42, CACNA2D4, FGF18, HSPA8, NF1, PRKCB, RPS6KA3, AKT3, MAP2K1, MKNK1, MAP3K8, CACNA2D1, RPS6KA4, RELA, CACNA1D, FGFR1, FGF7, PLA2G4C, ARRB1*	8.62 × 10^−09^
Wnt signaling pathway	miR-15b-5p, miR-145-5p, miR-299-5p, miR-197-3p, miR-185-5p	*CTNNBIP1, CAMK2D, DAAM2, LRP6, WNT7A, BTRC, VANGL1, CCND2, SMAD3, WNT2B, PPP2R5C, WNT5B, SKP1, PPP2R5A, PPP3CA, NLK, PLCB1, FZD4, SENP2, FZD10, CCND1, AXIN2, NFATC2, MAPK8, PPP2R1A, SIAH1, PRKCB, CXXC4, WNT3A, WIF1, WNT9B, NFATC3, PPP2R1B, TBL1XR1, CCND3*	1.06 × 10^−08^
p53 signaling pathway	miR-15b-5p, miR-145-5p, miR-299-5p, miR-197-3p, miR-185-5p	*CCNG1, ZMAT3, BAI1, RFWD2, CDK2, CCND2, PERP, CDK6, CHEK1, CCND1, SHISA5, SESN1, SIAH1, CCNE1, IGFBP3, SESN3, PTEN, PPM1D, CCND3*	2.88 × 10^−08^
Focal adhesion	miR-15b-5p, miR-126-3p, miR-145-5p, miR-299-5p, miR-197-3p, miR-185-5p	*ACTB, PDGFRA, MYLK4, SOS2, CRK, PAK2, COL24A1, PPP1CC, CCND2, ITGB6, PAK7, RAF1, BCL2, IGF1R, ZYX, VCL, ITGA1, PTK2, CCND1, MAPK8, FLNB, FLNA, PIK3R1, COL4A4, CDC42, PAK6, PRKCB, AKT3, MYLK3, MAP2K1, ITGA6, VEGFA, PTEN, KDR, ARHGAP5, MYLK, CCND3, ILK*	4.45 × 10^−06^

### A 2-miRNA signature has an independent prognostic value for MPM

**Figure 2:**
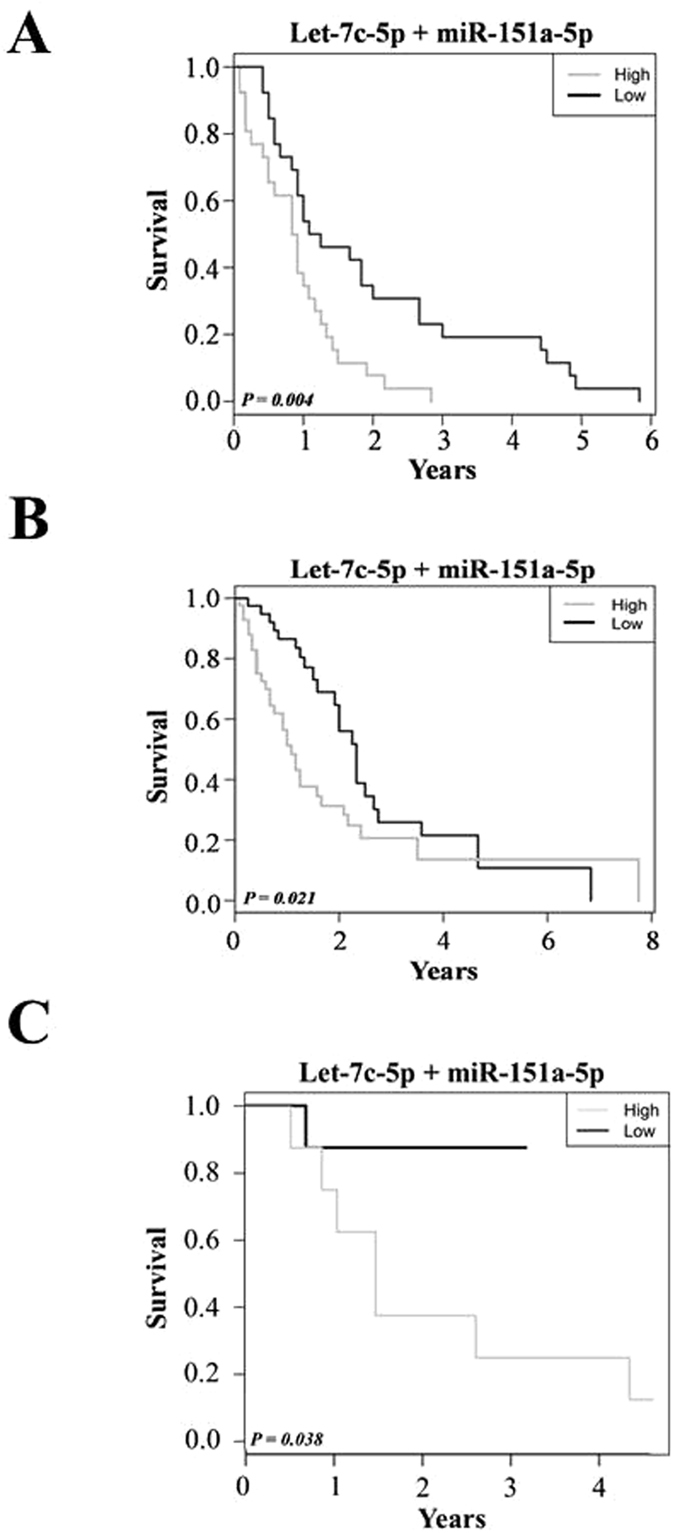
Kaplan-Meyer curves representing the correlation between 2-miRNA signature based on Let-7c-5p and miR-151a-5p expression levels and overall survival rates in MPM patients. High levels (grey line) of Let-7c-5p plus miR-151a-5p were associated with a significantly worse overall survival than low levels (black line) in patients recruited in the present study (**A**), in the TCGA mesothelioma dataset (**B**) and in an independent set of fresh frozen MPM (**C**) (*P* = 0.004, *P* = 0.021, and *P* = 0.038, respectively).

Finally, miRNAs from microarray analysis were also evaluated as potential prognostic biomarkers, considering only the 52 MPM patients for whom the survival data were available. We identified a 2-miRNA signature based on Let-7c-5p and miR-151a-5p expression levels. The OS and a risk score for each patient was calculated as explained in the materials and methods section. The OS rates were significantly lower in the patient group with the high-risk score (P = 0.004 by the log-rank test; Fig. 2A). We next sought to validate the risk score using the miRseq data from the TCGA (The Cancer Genome Atlas) mesothelioma dataset. When patients in validation cohort were stratified according to their risk score, the group with a low risk score had a significantly better prognosis (P = 0.021 for OS, log-rank test) than group with a high risk score (Fig. 2B). Similar results were obtained when assessing the 2-miRNA signature in association with prognosis in our cohort of 16 fresh frozen MPM (P = 0.038 for OS, log-rank test; Fig. 2C). Taken together, these results show that the classifier based on Let-7c-5p and miR-151a-5p expression levels has great potential as prognostic tool.

In order to ascertain whether the 2-microRNA signature indeed performs better than single miRNAs, we compared the performances of the 2-microRNA signature and the single miRNAs for OS by Akaike information criterion (AIC). The lowest AIC of the model is based on the two miRNAs taken together and it suggests its better performance than the single miRNAs in our cohort (307.1 vs 310.1 and 310.0) and in the TCGA cohort as well (402.3 vs 403.4 and 403).

The effect of risk score, gender, age and histotype on patient survival was further evaluated by multivariate Cox proportional hazard model on 52 MPM subjects from the microarray sample set. Histologic subtype and gender were not associated with survival in our sample set (P = 0.34 and P = 0.20, respectively), while a lower age at the diagnosis (<65 years old) was significantly associated with better prognosis (P = 0.001). In this model, risk score was an independent predictor of patient survival (P = 0.002).

Moreover, the effect of risk score and therapy on the prognosis was evaluated on 27 MPM subjects for whom the information regarding the received treatments was available. A non-symptomatic therapy (including surgery, chemotherapy or radiotherapy) was significantly associated with a better prognosis (P = 0.008), and the risk score was again an independent factor of patient survival (P = 0.027).

## Discussion

MPM is a very aggressive and heterogeneous tumour^8^. Its biology is still unclear and further studies are needed in order to discover new pathways potentially helpful in diagnosis, prognosis and therapy. MiRNAs were shown to play a key role in the pathogenesis of MPM^4^.

Here, we aimed to broaden the knowledge about miRNAs in MPM investigating the presence of differentially expressed miRNAs using a microarray platform. This approach allowed the identification of six miRNAs (miR-15b, miR-126, miR-145, miR-185, miR-197, and miR-299), three of which (i.e. miR-185, miR-197 and miR-299) have never been linked, to the best of our knowledge, to MPM carcinogenesis. These miRNAs have been shown to target specific pathways involved in cancer outcome, progression or therapy. For instance, miR-185 was reported to be down-regulated in hepatocellular carcinoma and to inhibit tumour growth by targeting the DNMT1/PTEN/Akt pathway^9^, commonly activated in MPM^10, 11^. It was shown to suppress tumour proliferation in triple-negative breast cancer by directly targeting E2F6 and DNTM1 and by simultaneously upregulating BRCA1^12^. A key anti-tumourigenic role of miR-185 was shown also in lung^13^ and gastric cancer^14^, modulating target mRNAs (i.e. *AKT1* and *TRIM29*, respectively) involved in the carcinogenic process. Another study revealed a role of miR-197 as tumour suppressor in mediating apoptosis in multiple myeloma cells by targeting *MCL1* ^15^, while in non-small cell lung cancer it contributed, when down-regulated, to chemoresistance through the miR-197/CKS1B/STAT3-mediated PD-L1 network^16^. MiR-299 was found down-regulated in prostatic cancer cell lines compared to normal cells^17^ and to play a role in autophagy inhibition by suppressing Atg5 in neurons^18^.

Moreover, we could replicate some of the results obtained by other authors^6, 7, 19–23^ who attempted to define a set of miRNAs differentially expressed between MPM and non-MPM mesothelial tissues (Table 2). Although we found 18 miRNAs already linked with MPM, the fact that some of the results were not in agreement with what previously reported should not be surprising. In fact, there are several reasons that could explain the only partial overlap with the literature data, i.e. different sample sources (FFPE vs fresh frozen tissues), different high-throughput platforms, different normalization techniques and, last but not least, the intrinsic heterogeneity of MPM. Nonetheless, we confirmed some interesting findings about three miRNAs (i.e. miR-15b, miR-126, and miR-145) that were shown to play a biological role in MPM. MiR-15b was down-regulated in MPM tumour specimens and was shown to inhibit growth of MPM cell lines^6^. It was also shown to act as tumour suppressor in liver cancer^24^ and its reduced expression was associated with chemo-resistance^25^ and poor prognosis^26^. MiR-126 has been extensively studied in MPM, where it was shown to have a proper biological activity both *in vitro* and *in vivo* ^5^ and a potential value as biomarker^22, 23, 27^. Many targets of miR-126 were experimentally validated in other cancer types, including *VEGF-A* ^28^, *ADAM9* ^29^, *Sox2* ^30^, *CRK*, and *PI3KR2* ^31^. The involvement of miR-126 in the regulation of angiogenesis was shown also in hepatocellular carcinoma, where it acts as tumour suppressor by targeting *LRP6* and *PIK3R2* ^32^. MiR-145 loss was reported to have pro-tumourigenic effects in MPM, via modulating clonogenicity, cell migration, and resistance to pemetrexed^7^. It was shown to be downregulated and to function as a tumour suppressor in other malignancies, namely nasopharyngeal carcinoma^33^, non-small cell lung cancer^34^, and bladder cancer^35^.

Altogether, the “miRNomic” approach allows highlighting a list of deregulated miRNAs, which could act as tumour suppressor or oncogene according to their target mRNAs. This strategy partially overcomes the limitation of the transcriptomics, which gives information about the dysregulated transcripts, but does not provide reliable data regarding their protein products, since mRNA levels often do not correlate with protein contents^36^. Indeed, miRNAs target analysis could help in the identification of a pattern of proteins possibly deregulated. Thus, following an *in silico* approach here we proposed novel targets for MPM, namely *GOLGA1*, *GOLGB1*, *PTPN9*, *PSMD1*, *TNRC6A*, *MPLZ1*, *PRKAR2A*, *CCNE1* and *VEGFA*.

Once the protein targets will be validated as deregulated in MPM, novel therapeutic strategies could be undertaken using specific inhibitors. Following this concept, PSMD1 and VEGFA are among the most suitable candidate to be investigated. *PSMD1* encodes one of the regulatory subunits of the proteasome complex and it was reported to be up-regulated in MPM^37^. It could suggest an unexplored therapeutic strategy for MPM, since the proteasome inhibitor Bortezomib has already shown to be effective in the treatment of multiple myeloma and mantle cell lymphoma^38^. *VEGFA* encodes the vascular endothelial growth factor A and it is reported to be up-regulated in mesothelioma^39^. The treatment with bevacizumab, a monoclonal antibody that blocks angiogenesis by inhibiting VEGF-A, in combination with pemetrexed plus cisplatin showed encouraging results in the therapy for MPM in a recent randomized phase III trial^40^.

A further aim of this study was to evaluate how miRNAs deregulation performs as a prognostic biomarker. Previous works highlighted the usefulness of miRNAs as prognostic biomarkers in MPM^20, 23, 41, 42^. While the earlier studies suggested a prognostic value for single miRNAs (miR-29c*^41^, miR-17-5p and miR-30c-5p^20^), subsequent works attempted to increase the accuracy of the survival analysis using a combination of multiple miRNAs (four-miRNA classifier^23^ or six-miRNA score^42^). In line with this approach, we proposed here a 2-miRNA signature (Let-7c-5p plus miR-151a-5p) relating to overall survival, whose prognostic value was also validated in the TCGA mesothelioma dataset and in an independent set of fresh frozen MPM samples. Our study showed some discrepancies compared to the literature data, where general poor overlap among studies could be at least partially ascribed to different microarray platforms (for example, miR-29c* and miR-652-3p probes were not included in the Nanostring platform), to subtle different statistical analyses in the calculation of the survival curves and, as acknowledged before, to the intrinsic heterogeneity of MPM. Nonetheless, our results were consistent with data provided recently about an eight-miRNA signature in bladder cancer, reporting an association between a high expression of Let-7c-5p and a poor outcome^43^. At a similar extent, high levels of miR-151 correlated with adverse effects on survival rate in prostate cancer^44^. Interestingly, both these miRNAs were reported to be associated with dysregulated metabolic conditions. In fact, Let-7c-5p plays a protective role against cerebral ischemia injury in mice^45^, while miR-151a-5p regulates energy metabolism reducing ATP production via mitochondrial dysfunction^46^. We have already stressed the role of mitochondrial abnormalities in MPM^47^, and we believe that hypoxia plays a crucial role in the resistance of MPM to conventional therapies. Let-7c-5p is reported to be involved in regulation of hypoxia^48, 49^, and can be considered members of the so-called “HypoxamiRs”, a group of miRs modulated by hypoxic conditions, involved in EMT and chemo-resistance and associated with worse prognosis^50^. Other evidences showed how miR-151-5p overexpression is linked with energy metabolism derangement and suggested a link with hypoxia^51^. This could explain their association with the overall survival that we observed here, since their high expression could promote the hypoxic microenvironment very well known to activate many signalling pathways involved in tumour initiation, progression and maintenance of MPM^52, 53^.

Although we provided overall reliable data about miRNA dysregulation in MPM, there are some limitations to the present study. First, we acknowledge a partial lack of clinical information for several patients since the sample collection was performed in different hospitals with different capabilities in retrieving old information from their records. Thus, the inclusion of more and more complete demographic details in the analyses for the identification of miRNA with diagnostic value could have improved the quality of the data provided. This limitation is particularly relevant in the prognostic calculations for the 2-microRNA signature, which were then done on relatively small sample numbers (n = 52, and information about relevant treatment was available only for 27 of them). Regarding the role of therapeutic treatments as prognostic variable, we acknowledge that our analysis was unable to discriminate among different treatments, since we simply grouped individuals in non-symptomatic vs symptomatic therapy in order to allow a sufficient statistical power in the multivariate analysis.

Despite this partial lack of clinical information, the baseline demographic features (age, gender and histological subtype) did not show any statistically significant difference between the cohorts with versus without survival data (see Supplementary Table 5 for further details), suggesting that the 2-miRNA prognostic signature could be applied to our entire sample set. Moreover, as explained in Table 4 and below in the materials and methods section, sufficient RNA could not be isolated from all samples, reducing the number of samples available for analysis. However, comparison of baseline characteristics of patients with and without RNA showed that there were no major differences between the respective groups (see Supplementary Table 5 for further details), thus the samples that we actually analysed were representative of a more general population.

**Table 4:** Demographic characteristic of the FFPE samples collected for the microarray study (n = 105 MPM and n = 10 normal pleural tissue).

Variables	Tot MPM Cohort (n = 105)				Tot non-MPM Cohort (n = 10)
	Patients with RNA (n = 96)		Patients without RNA (n = 9)		Patients RNA (n = 10)
	Patients with demographic information (n = 66)^&^		Patients with demographic information (n = 7)^&^		Patients with demographic information (n = 10)
	With survival data (n = 52)	Without survival data (n = 14)*	With survival data (n = 7)	Without survival data (n = 0)	
**Mean age** (range)	65.5 (41–85)	69 (68–70)	72.8 (67–80)	N/A	72.4 (57–84)
**Gender**					
Male	35 (67%)	2 (100%)	5 (71%)	N/A	7 (70%)
Female	17 (33%)	0 (0%)	2 (29%)	N/A	3 (30%)
**Histological subtype**					
Epithelioid	43 (83%)	14 (100%)	4 (57%)	N/A	
Biphasic	8 (15%)	0 (0%)	3 (43%)	N/A	
Sarcomatoid	1 (2%)	0 (0%)	0 (0%)	N/A	
**Median OS** (months) (range)	11.5 (1–70)	N/A	12 (8–22)	N/A	
**Therapy** ^**$**^					
Non-symptomatic^£^	13				
Symptomatic	14				

^&^Total number of MPM patients with partial/total demographic information is 73 (66 + 7); *For this group the information about gender and age was available only for 2/14 patients; N/A = not available; ^$^Data was available only on 27/105 samples, all falling within the patients group with survival data; ^£^Non-symptomatic therapy included pleurectomy with decortication (P/D) (n = 3), P/D + chemotherapy (n = 3), pleuropneumonectomy (n = 1), pleuropneumonectomy + chemotherapy (n = 3), pleuropneumonectomy + chemotherapy + radiotherapy (n = 2), pleuropneumonectomy + radiotherapy (n = 1).

## Conclusion

In conclusion, with the present work we showed that the pattern of miRNAs expression is highly deregulated in MPM, as reported in many other tumour types. We also suggest that alterations in miRNA expression could modify cell pathways regulation and could allow the discovery of new druggable targets, following appropriate experimental validation. We also identified a 2-miRNA signature as useful biomarker for prognosis in MPM clearly addressing toward a role of metabolism in MPM aggressiveness. Patients stratified in high risk group, by our classifier, might benefit either of more targeted and aggressive cures or therapies targeting metabolic reprogramming in order to improve their outcome. Further studies on the miRNA target-genes are needed to better ascertain their fruitful exploitation as therapeutic targets or as diagnostic and prognostic biomarkers of MPM.

## Materials and Methods

For this work, according to the Helsinki declaration, volunteers gave informed consent for the research. The study was approved by the institutional ethical committee of the University Hospital of Pisa. All the experimental protocols were carried out according to the relevant guidelines, as explained in details in the sections below.

### Tissue collection

A consecutive series of 105 MPM patients provided corresponding 105 formalin-fixed paraffin-embedded MPM tissues. The MPM FFPE samples were biopsies collected from 2005 to 2009 in collaboration with the units of Thoracic Surgery and Occupational Medicine at the University Hospital of Pisa, together with the Hospital of Brescia, the Hospital of Venice (ASL 12), and the IRCCS AOU San Martino-IST in Genoa. All institutions providing the tissues followed the same protocol of sample collection and tissue preparation. The MPM diagnosis was issued from pathologists, following the standard clinical routine that was based on microscopic inspection and immuno-histochemical analyses of slides with antibodies to detect a panel of biomarkers (CK5/6, calretinin, vimentin, CK-Pan, EMA, TTF1, BerEP4, CEA). Pleural tissue specimens for the control sample set (n = 10) were collected in the University Hospital of Novara from individuals with chronic pleuritis (n = 6) and mesothelial hyperplasia (n = 4). Samples were selected for the microarray study only when RNA of sufficient quality and quantity was available after extraction from FFPE tissues. Nine MPM FFPE samples among the 105 available did not meet the quality criteria either in the RNA extraction or in the microarray run. Baseline characteristics of the 105 MPM patients (including the 96 samples used in the study) and the 10 non-MPM individuals are provided in Table 4. We used the full cohort of 96 RNA samples with known diagnosis of MPM to identify miRNAs with a potential diagnostic value, i.e. miRNAs that were differentially expressed between MPM and normal pleural samples. Among the 96 MPM patients used in this study, partial/total demographic information was available for 66 individuals. The survival time, calculated from the date of diagnosis, was available for 52 patients, who were then also employed to identify miRNAs with a potential prognostic value.

For the validation analysis, 22 MPM biopsies were collected during thoracoscopy in collaboration with the units of Thoracic Surgery and Occupational Medicine at the University Hospital of Pisa (Italy) before any treatment. About 70% of MPM patients had an ascertained positive history of exposure to asbestos, and approximately half of them had also a positive cigarette smoking history. Control tissues (20) were normal pleura from patients who underwent surgery for early-stage lung cancer (6 lung adenocarcinomas and 14 lung squamous cell carcinomas). Pleural specimens were collected far from the tumour site, eye-inspected by surgeons and analysed by pathologists in order to collect a small portion of pleura not containing evidence of lung cancer spread. All samples were fresh frozen, stored in the RNA later reagent (Qiagen, Hilden, Germany) and placed at −80 °C right after the collection. Following the same criteria of the microarray study, samples were selected for the validation study only when RNA of sufficient quality and quantity was available after extraction from fresh frozen tissues, and for this reason six and three RNA samples were excluded among the MPM and non-MPM subgroup, respectively. Baseline characteristics of the 42 MPM and non-MPM samples and the subset of 33 patients used in this validation study are provided in Table 5. The validation of the 2-miRNA signature was performed on the full set of 16 MPM with RNAs as the survival time was available for all of them.

**Table 5:** Demographic characteristic of the fresh frozen samples collected for the validation study (n = 22 MPM and n = 20 normal pleural tissue).

Variables	Tot MPM Cohort (n = 22)		Tot non-MPM cohort (n = 20)	
	Patients with RNA (n = 16)	Patients without RNA (n = 6)	Patients with RNA (n = 17)	Patients without RNA (n = 3)
**Mean age** (range)*	63.3 (40–77)	77 (64–87)	73.5 (59–85)	67.6 (63–72)
**Gender**				
Male	13 (81%)	5 (83%)	10 (59%)	2 (67%)
Female	3 (19%)	1 (17%)	7 (41%)	1 (33%)
**Histological subtype**				
Epithelioid	11 (69%)	4 (66%)		
Biphasic	4 (25%)	0 (0%)		
Sarcomatoid	1 (6%)	2 (33%)		
**Median OS** (months) (range)	19.5 (6–53)	14.5 (1–41)		

*Statistically different between patients with RNA and patients without RNA in the MPM subgroup (defined as p < 0.05 in Student’s *t*-test).

### Microarray Study

#### RNA extraction

Total RNAs were isolated from formalin-fixed paraffin-embedded tissues (FFPE) using Recover All kit (Thermo Fisher Scientific, Waltham, USA), following manufacturer’s protocol. About 100 ng of total RNA per samples was then processed with the Human miRNA Expression Assay from NanoString nCounter system (nanoString, Seattle, Washington, USA).

#### nanoString nCounter profile analysis

RNAs were processed by the nanoString nCounter system (nanoString, Seattle, Washington, USA) in the Nucleic Acid Shared Resource of The Ohio State University. The miRNA panel detects 800 endogenous miRNAs, five housekeeping transcripts [actin beta (NM_001101.2), beta-2 microglobulin (NM_004048.2), GAPDH (NM_002046.3), RPL19 (NM_000981.3), and RPLP0 (NM_001002.3)], six positive and eight negative controls (proprietary spike-in controls).

#### Data analysis, statistical methods, and figures

Raw expression data, which are proportional to copy number, were log-transformed and normalized by the quantile method after application of a manufacturer-supplied correction factor. Data were filtered to exclude features below the detection threshold (defined for each sample by a cutoff corresponding to ~twice standard deviation of negative control probes plus the means) in at least half of the samples. Using R/Bioconductor and the filtered dataset, linear models for microarray data analysis (limma) were employed with a contrast matrix for comparing normal vs tumour. Raw data that were above background (analysis not shown), as well as the corresponding quantile-normalized data, were also imported into MultiExperiment Viewer. Data processing and analysis were conducted using BRB-ArrayTools^54^, the MultiExperiment Viewer^55^, and R/BioConductor packages including limma^56^. The analysis of raw data was performed with nSlover Analysis Software provided by NanoString Technologies®. Data were collected as NanoString RCC files and provided as an Excel spreadsheet. P values were used to rank miRNAs of interest, and correction for multiple comparisons was done by the Benjamini-Hochberg (BH) method^57^.

Survival analysis was carried out using Kaplan–Meier curves on 52 MPM patients. To generate a risk score, we adopted a previously developed strategy^58^ that uses the Cox proportional hazards regression analysis to identify the miRNA associated with survival. MiRNAs whose expression was statistically significantly associated with survival at P ≤ 0.05 were selected by fitting Cox proportional hazards models to each miRNA in the training data. A Cox proportional hazards model was built using the principal components calculated from the statistically significant miRNA list. The OS and a risk score for each patient was calculated by multiplying the expression level of the 2 miRNAs by their corresponding Cox regression coefficient and summing the resulting values (risk score = sum of each Cox coefficient of miRNA x expression value of the corresponding miRNA). Thus, patients were dichotomized into groups at high or low risk using the 50th percentile (median) cutoff of the risk score as the threshold value. In order to validate our prognostic signature, the TCGA mesothelioma dataset (validation set/cohort, http://cancergenome.nih.gov/cancersselected/Mesothelioma) was used an independent set. MiRNA expression data for 83 tumours from patients with MPM were downloaded and the survival analysis was carried out applying directly the Cox coefficient derived by our cohort.

The performances of the 2-microRNA signature and single microRNAs for OS were compared by AIC, a measure of global fit that offers a relative estimate of the information lost when a given model is used to represent the process that generates the data, where low AIC values indicate better fit^59^.

The effect of other known prognostic factors on the survival of 52 MPM patients (histological subtype, gender and age) was also tested together with the risk score in a multivariate Cox proportional hazard model. Moreover, information about therapy was available for 27 patients with survival data, thus we performed another multivariate analysis considering the 2-miRNA risk score and the treatment (symptomatic therapy, n = 13, vs non-symptomatic therapy, n = 14) as prognostic variables.

### Validation study

#### RNA isolation and cDNA synthesis

RNA from individual biopsies was purified by using Tri-Reagent method (Sigma Aldrich Corp. St Louis, MO, USA). In order to remove any contaminating genomic DNA, the extracted RNA was treated with DNase (Sigma Aldrich Corp. St Louis, MO, USA) and its concentration was determined by spectrophotometer (SmartSpec 3000, Bio-Rad Laboratories, Hercules, CA). The integrity and purity of total RNA were further verified by electrophoresis on ethidium bromide agarose gel, inspecting the 18 and 28 S ribosomal RNA bands. RNAs which did not meet the quality criteria (i.e. good concentration and integrity) were excluded from the analyses. The reverse transcription (RT) was performed using the TaqMan® MicroRNA Reverse Transcription Kit (Life Technologies, Monza, Italy) using stem-loop specific miRNA primers starting from 100 ng of total RNA.

#### Selection of reference miRNAs for RT-qPCR and RT-qPCR

In order to perform accurate RT-qPCR measurements, we tested a panel of possible reference miRNAs/short RNAs commonly used in miRNA expression analyses. The stability of each of them was measured by *geNorm* ^60^ and, according to the average M and the pair-wise variation values, U6, RNU48, RNU44 were found stable and were employed as reference miRNAs in all the experiments. Pre-designed TaqMan probes (Life Technologies, Monza, Italy) were employed. For the TaqMan assay, the reaction mixture consisted of 1.33 μl of cDNA template, 7.67 μl of deionized H_2_O, 1 μl of specific TaqMan Assay probe and primer mixture, and 10 μl of TaqMan® Universal Master Mix II NO UNG (Life Technologies, Monza, Italy). The thermal cycling conditions were: 10 min at 95 °C followed by 40 cycles made of 15 s at 95 °C and 60 s at 60 °C. TaqMan ID assays are reported in Supplementary Table 5. Each sample was run in triplicate, and the quality control of the derived expression values was performed according to MIQE guidelines^61^.

#### Statistical analyses

For validation analysis, miRNAs expression levels from MPM samples were compared to control tissue samples by applying the two-sided Mann–Whitney–Wilcoxon test. The adjustment for multiple testing was performed by using Bonferroni’s correction, thus the novel statistical significance threshold was 0.01. Data analysis was produced by the software Statgraphics Centurion XV (StatPoint, Inc.). Moreover, in order to confirm the 2-miRNA signature associated with prognosis, the OS and a risk score for each patient was calculated as previously explained for the microarray set of analyses.

### *In silico* analyses

*In silico* prediction of the potential target mRNAs was performed for each interesting miRNA using DIANA-microT-CDS tool^62^, with threshold set at 0.6. DIANA-miRPath^63^ was employed to perform the over-representation analysis using the differentially expressed miRNAs as input list. DIANA-miRPath allows the identification of miRNA-target genes, either predicted (the “microT-CDS” mode) or experimentally validated (the “TarBase” mode). The option “Union Set”, that collects all genes targeted by at least one selected miRNAs, was applied for the over-representation analysis.

## Electronic supplementary material

suppl_material_wordfiles

Suppl Table 1

Suppl Table 2

Suppl Table 3

Suppl Table 4
