# Supplementary material for: Deregulation of miRNAs in malignant pleural mesothelioma is associated with prognosis and suggests an alteration of cell metabolism
Source: Sci Rep. 2017 Jun 9;7:3140. doi: 10.1038/s41598-017-02694-0 (PMC5466648; doi:10.1038/s41598-017-02694-0)
Supplement: Supplementary file 1 — suppl_material_wordfiles [file 41598_2017_2694_MOESM1_ESM.doc]

**SUPPLEMENTARY MATERIAL FILES**

**Deregulation of miRNAs in malignant pleural mesothelioma is associated with prognosis and suggests an alteration of cell metabolism**

**Authors:**

Chiara De Santi^1, †^, Ombretta Melaiu^2, †^, Alessandra Bonotti^3, †^, Luciano Cascione^4^, Gianpiero Di Leva^5^, Rudy Foddis^6^, Alfonso Cristaudo^6^, Marco Lucchi^7^, Marco Mora^8^, Anna Truini^8^, Andrea Tironi^9^, Bruno Murer^10^, Renzo Boldorini^11^, Monica Cipollini^12^, Federica Gemignani^12^, Pierluigi Gasparini^13^, Luciano Mutti^5^ and Stefano Landi, Associate Professor^12,*^.

**Affiliation:**

^1^Respiratory Research Division, Department of Medicine, Education and Research Centre, Royal College of Surgeons in Ireland, Beaumont Hospital, Dublin 9, Ireland

^2^Immuno-Oncology Laboratory, Department of Paediatric Haematology/Oncology, Ospedale Pediatrico Bambino Gesù, Viale di S. Paolo 15, 00146 Rome, Italy

^3^Preventive and Occupational Medicine, University Hospital of Pisa, Pisa, Italy

^4^Lymphoma and Genomics Research Program, Institute of Oncology Research, Bellinzona, Switzerland

^5^School of Environment and Life Sciences, University of Salford, Manchester, United Kingdom.

^6^Department of Translational Research and of new Technologies in Medicine and Surgery, University of Pisa, Pisa, Italy

^7^Division of Thoracic Surgery, Cardiac and Thoracic Department, University of Pisa, Pisa, Italy ^8^IRCCS H. San Martino-IST Genova, Genova, Italy

^9^Section of Anatomic Pathology, Oncology and Experimental Immunology, Department of Molecular and Translational Medicine, University of Brescia, Brescia, Italy

^10^Azienda ULSS 12 Veneziana, Venice, Italy

^11^Department of Health Sciences, School of Medicine, University Hospital Maggiore della Carità, Novara, Italy

^12^Department of Biology, University of Pisa, Pisa, Italy

^13^Department of Molecular Virology, Immunology and Medical Genetics, Ohio State University Wexner Medical Center and Comprehensive Cancer Center, Columbus, Ohio, USA

^*^Correspondence to: Stefano Landi, Department of Biology, University of Pisa, Via Derna, 1, 56126 Pisa, Italy. Tel.: +39 050 2211528; Fax: +39 0502211527, slandi@biologia.unipi.it

^†^These authors contributed equally to the manuscript

**SUPPLEMENTARY MATERIALS**

**Supplementary Table 1**. List of the raw and normalised expression data for each sample for each miRNA measured by the Nanostring nCounter. Information about the quantity and the quality of the RNA samples is also provided in the “Nanodrop QC” worksheet.

**Supplementary Table 2**. In this table for each miRNA we reported the p-value of the comparison between MPM and non-MPM controls before (“raw”) and after (“BH”) Benjamini-Hochberg correction. The P-value and the statistic value (D) for the Kolmogorov-Smirnov (KS) test is also shown, as further non-parametric test to compare miRNAs expression between MPM and non-MPM controls. We also showed the extent of the differential expression (“Differential Expression”), the median of the expression level of MPM (“Median_MPM”) and non-MPM controls (“Median_Normal”), and the values of the expression level of each miRNA for each sample.

**Supplementary Table 3**. List of the raw and normalised Ct values for each sample for each miRNA measured by qRT-PCR with TaqMan MicroRNA Assays in the validation study. Information about the quantity and the quality of the RNA samples is also provided in the “Spectophotometer_RNA” worksheet.

**Supplementary Table 4**. List of the top 50 predicted targets of the six highlighted miRNAs obtained with DIANA-microT-CDS. They are ranked according to miTG score, and both Transcript and Gene Id are reported.

**Supplementary Table 5**. List of TaqMan assay IDs of selected miRNAs undergone to validation and short RNAs used as reference.

**Supplementary Table 5.** Comparison of baseline demographic features in different subgroups of patients (MPM and non-MPM) enrolled in this study.

|  | **Comparisons** | **Age*** | **Gender^$^** | **Histological subtype^$^** |
| --- | --- | --- | --- | --- |
| **Microarray study** | MPM patients with RNAs with (n=52) vs without (n=14) survival data | P=0.70 | P=0.33 | P=0.25 |
|  | MPM patients with (n=96) or without (n=9) RNAs^£^ | P=0.12 | P=0.87 | P=0.094 |
|  | MPM (n=105) vs non-MPM (n=10) patients | P=0.13 | P=0.94 | N/A |
| **Validation study** | MPM patients with (n=16) or without (n=6) RNAs | **P=0.007** | P=0.91 | P=0.146 |
|  | Non-MPM patients with (n=17) or without (n=3) RNAs | P=0.17 | P=0.80 | N/A |
|  | MPM (n=22) vs non-MPM (n=20) patients | P=0.06 | P=0.12 | N/A |
| MPM (microarray study, n=105) vs MPM (validation study, n=22) | | P=0.91 | P=0.24 | P=0.12 |
| Non-MPM (microarray study, n=10) vs non-MPM (validation study, n=20) | | P=0.94 | P=0.59 | N/A |
| Total samples (microarray study, n=115) vs total samples (validation study, n=42) | | P=0.30 | P=0.79 | N/A |

*comparisons of age distributions: since the age values were distributed normally in all the subgroups employed in the comparisons (D'Agostino & Pearson normality test P>0.05; when the sample set was small (n<10), the Shapiro-Wilk normality test was used instead), the differences were assessed with Student’s *t*-test; **^$^**comparisons of gender and histological subtype distributions: the differences were assessed with Chi-square (χ^2^) test. ^£^In this comparison, demographic info was available for 66/96 MPM patients with RNAs and 7/9 MPM patients without RNAs. All the statistical analyses were performed with Statgraphics Centurion XV (StatPoint, Inc.). N/A=not applicable

**Supplementary Table 6.** List of TaqMan assay IDs of selected microRNAs undergone to validation and short RNAs used as reference.

| **microRNA/ short RNA** | **TaqMan assay ID** |
| --- | --- |
| miR-337-3p | 002157 |
| miR-185-5p | 002271 |
| miR-485-3p | 001277 |
| miR-197-3p | 000497 |
| miR-299-5p | 000600 |
| let-7c-5p | 000379 |
| miR-151a-5p | 002642 |
| U6 siRNA | 001973 |
| RNU44 | 001094 |
| RNU48 | 001006 |
